# A comprehensive map of genome-wide gene regulation in *Mycobacterium tuberculosis*

**DOI:** 10.1038/sdata.2015.10

**Published:** 2015-03-31

**Authors:** Serdar Turkarslan, Eliza J R Peterson, Tige R Rustad, Kyle J Minch, David J Reiss, Robert Morrison, Shuyi Ma, Nathan D Price, David R Sherman, Nitin S Baliga

**Affiliations:** 1 Institute for Systems Biology, Seattle, Washington 98109, USA; 2 Seattle Biomedical Research Institute, Seattle, Washington 98109, USA; 3 Interdisciplinary Program of Pathobiology, Department of Global Health, University of Washington, Seattle, Washington 98195, USA; 4 Department of Chemical and Biomolecular Engineering, University of Illinois, Urbana, Illinois 61801, USA; 5 Molecular and Cellular Biology Program, University of Washington, Seattle, Washington 98195, USA; 6 Departments of Biology and Microbiology, University of Washington, Seattle, Washington 98195, USA; 7 Lawrence Berkeley National Lab, Berkeley, California 94720, USA

**Keywords:** Bacterial genes, High-throughput screening, Regulatory networks, Transcriptomics

## Abstract

*Mycobacterium tuberculosis* (MTB) is a pathogenic bacterium responsible for 12 million active cases of tuberculosis (TB) worldwide. The complexity and critical regulatory components of MTB pathogenicity are still poorly understood despite extensive research efforts. In this study, we constructed the first systems-scale map of transcription factor (TF) binding sites and their regulatory target proteins in MTB. We constructed FLAG-tagged overexpression constructs for 206 TFs in MTB, used ChIP-seq to identify genome-wide binding events and surveyed global transcriptomic changes for each overexpressed TF. Here we present data for the most comprehensive map of MTB gene regulation to date. We also define elaborate quality control measures, extensive filtering steps, and the gene-level overlap between ChIP-seq and microarray datasets. Further, we describe the use of TF overexpression datasets to validate a global gene regulatory network model of MTB and describe an online source to explore the datasets.

## Background & Summary

Tuberculosis is a top global health concern, killing more people than any infectious disease except HIV^1^. Our understanding of the disease and its infectious agent, *Mycobacterium tuberculosis* (MTB) has progressed tremendously since the discovery of MTB more than 100 years ago. However, our methods for diagnosis, prevention and treatment for MTB infection are outdated and call for new approaches. The underlying complexity of MTB infection still poses a challenge in identifying principles that drive the onset of infection and disease progression. This complexity combined with the dynamic adaptations of MTB to diverse environmental conditions from latency to active disease states demands systems level approaches to decipher the molecular mechanisms of MTB infection.

In recent years, we have seen the development of a substantial infrastructure to enable systems biology, including the development of important tools for data generation, analysis and modeling. A number of studies for systematic profiling of gene expression, proteins, lipids and metabolites have generated new insights for the molecular mechanisms of MTB pathogenicity^2–7^. In addition, a global survey of *in vivo* and *in vitro* essentiality for the MTB genome provided invaluable insights for systems biology of TB^8,9^.

*In vivo*, MTB encounters a number of strong stimuli including hypoxia and nutrients that are hypothesized to trigger a coordinated response orchestrated by regulatory readjustments. Several previous studies have probed signatures of these regulatory adjustments using gene expression data from MTB subjected to various environmental stresses^4,10–13^. Similarly, we have recently made significant progress in the systems analysis of MTB by developing a high-throughput system for global analysis of transcription factor binding and gene expression^2^. In order to assemble a comprehensive map of DNA-binding events, we targeted all TFs in the MTB genome and built episomally expressed FLAG-tagged constructs under the control of a mycobacterial tetracycline-inducible promoter. In parallel, we cloned and conditionally overexpressed these transcription factors (TFs) under the control of the same inducible promoter providing a systematic toolbox for a genome-wide query of binding events and the possible associated gene expression consequences. In our initial analysis using this toolbox, we were able to map DNA-binding sites and transcriptional consequences for 50 MTB TFs (23% of 214 TFs of MTB)^2^.

**Figure 1:**
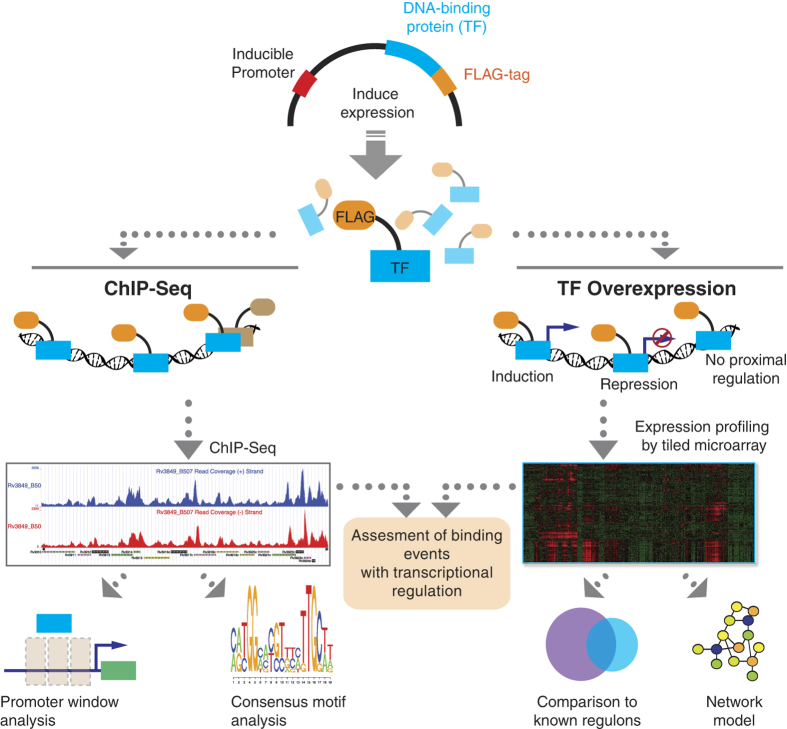
ChIP-Seq and TFOE Analysis Workflow. All putative DNA-binding proteins in MTB genome were cloned into expression vector with FLAG-tag under the control of inducible promoter. After induction of expression, either chromatin immunoprecipitation followed by sequencing or transcriptional profiling by using high-density tiling arrays was performed for each TF. For ChIP-seq experiments, confident binding events across genome were identified after analysis and filtering of read pileups as described in Methods section. These binding events were further investigated with respect to transcription start sites and compared to expression consequences in TFOE dataset. Consensus DNA binding motifs were also identified. For TFOE experiments, differentially expressed genes were identified by microarray analysis. Differential expression signatures were used to build a transcriptional network model. Moreover, TFOE-derived regulatory influences were compared to 12 existing regulons as a validation.

In this study, we further expand the ChIP-seq and TF overexpression data to 206 TFs to build a foundation for mapping the detailed regulatory landscape of MTB. These two datasets are summarized in two publications; the ChIP-seq data are described in Minch *et al.* ^14^ and the transcription factor overexpression data are described in Rustad *et al.* ^15^ The Minch *et al.* study extended the high-throughput ChIP-seq assay to 206 TFs. Genes for 206 proteins annotated as DNA-binding proteins were cloned under the control of an inducible promoter and FLAG-tagged for immunoprecipitation. DNA-binding events were determined after computational analysis of the DNA sequencing reads for each strain following immunoprecipitation. This analysis revealed regulatory binding events for more than 80% of the predicted MTB TFs. Binding profiles were filtered to identify high-confidence binding sites and to determine TF-binding consensus motifs. More than 1,500 of these binding events were associated with proximal gene regulation after filtering through the TF overexpression (TFOE) dataset (Fig. 1 and Supplementary Table 1). In order to associate TF binding with regulatory consequences, Rustad *et al.* ^15^ investigated the transcriptional signatures of the same set of 206 TF using high-density tiling microarrays and identified 9,335 changes associated with TF overexpression (Fig. 1 and Supplementary Table 2). Functional roles were assigned for many of these TFs based on the analysis of the expression changes in the context of target genes and gene ontology groupings. Furthermore, TFOE data were used to guide the metabolic network model of MTB for predicting the growth rates for TFOE strains.

Here, we describe a substantial baseline MTB regulatory network data based on large-scale ChIP-seq and expression analyses. We provide detailed definitions of these two datasets, associated analysis workflows, integration with an independent gene regulatory network model^16^ and description of an accessible web-based resource for further data access and exploration. The complementary large-scale data sets and predictive models generated in this study will generate community-wide resources to catalyze novel approaches to study TB.

## Methods

### Selection of transcription factors for analysis

178 genes annotated as TFs plus 13 genes annotated as sigma factors were selected from Tuberculist^17^. We excluded 4 genes (a methlytransferase; Rv0560c and three MoxR orthologs; Rv1479, Rv3164c, and Rv3692) that do not contain any DNA-binding domain. This list was expanded by adding 27 additional genes based on presence of transcriptional regulation-relevant COG domain^18^. Our final set of TFs included 214 genes, 206 of which we were able to clone for subsequent analyses (Supplementary Tables 1 and 2).

### Construction of expression vectors and strains

Vector construction for ChIP-seq experiments was performed as described in Minch *et al.* ^14^ Briefly, putative DNA-binding genes were identified and either selected from the Gateway Entry Clone library, if available, or amplified by PCR to include the Gateway recombination sequences. In total, 206 genes were cloned into the anhydrotetracycline (ATc)-inducible, *E. coli*-mycobacterial episomal shuttle vector with an N- or C-terminal FLAG epitope tag for immunoprecipitation. All culturing for ChIP was performed in Middlebrook 7H9 media (with ADC supplement and 0.05% Tween80) at 37 °C under aerobic conditions with hygromycin B (50 μg ml^−1^) in the media. Expression was induced for 18 h using 100 ng ml^−1^ ATc with cultures at a starting OD600 of 0.35.

### Chromatin immunoprecipitation

A detailed protocol along with all buffer recipes for chromatin immunoprecipitation is given in Minch *et al.* ^14^ Briefly, cultures were cross-linked with 1% formaldehyde at room temperature for 30 min with constant agitation. 250 mM glycine was added to stop cross-linking and cells were immediately pelleted, washed with PBS buffer containing protease inhibitor cocktail and resuspended in ChIP buffer. Cells were lysed mechanically using Lysing Matrix B tubes and 3 rounds of bead beating at max speed for 30 s with cooling on ice between treatments. Supernatants in ChIP Buffer 1 were subjected to ultrasonication for 16 min to obtain approximately 200-bp sheared chromatin fragments. After sonication, the buffer was changed to IPP150 and samples were incubated with 10 μg M2 anti-FLAG antibody at 4 °C overnight to initiate immunoprecipitation of FLAG-tagged proteins. FLAG-tagged proteins were collected by using incubation with protein G-coupled agarose beads followed by centrifugation and collection of pellets. Sample pellets were washed 5 times with IPP150 buffer and protein complexes were eluted in two steps. First, protein-bead complexes were incubated in elution buffer 1 for 15 min at 65 °C followed by centrifugation. Then, samples were treated with TE, pH 8.0 and 1% SDS for 5 min at 65 °C followed by centrifugation. Pooled supernatants were incubated with 1 mg/ML Pronase for 2 h at 42 °C followed by 9 h at 65 °C to reverse the cross-links. Immunoprecipitated DNA was further purified using QiaQuick PCR purification columns. Samples were sequenced using standard Illumina protocols producing 30–50 million 40-bp single-end reads. Replicate experiments were performed for some but not all the samples. Sample names of replicate experiments for a given TF are encoded as TF Name _ BXXX where BXXX indicates experiment identifier.

### ChIP-Seq read alignment and peak calling

Reads were aligned to the reference genome using the Bowtie 0.12.7 algorithm^19^ followed by peak calling with an in-house algorithm as described in Minch *et al.* ^14^ Read pileups were converted into wiggle tracks and peaks were probed by searching for local maxima. Gaussian or Gumbel model distributions that are best fit for the aligned reads were determined using nonlinear least squares optimization. Scores from 0 to 1 were assigned to each peak based on the width, height and deviation from the local maxima. A combined peak was created by combining forward and reverse cumulative wiggle tracks, and final binding score was performed with the addition of score values for separation and relative heights of forward and reverse peak center points. Ten control experiments were used as a negative control set for calculating significance *P*-values for each peak as described in the technical validation section. After scoring peaks from each individual experiment, peaks data for transcription factors with multiple experimental replicates were consolidated, and peaks significant *P*-values from each transcription factor were extracted and assembled into a combined network. Codes for ChIP-seq peak calling and consolidation are available at http://networks.systemsbiology.net/mtb/software. Please see Supplementary Fig. 10 in Minch *et al.* ^14^ for the complete ChIP-seq peak-calling workflow.

### Microarray analysis

RNA samples were isolated from each TFOE expression strain as described in Rustad *et al.* ^15^ Gene expression was measured using custom Nimblegen tiling arrays with 100-bp spacing. Labeling of RNA with Cy dyes and hybridization to array slides was performed as described previously^4^. Expression levels for all the probes were corrected for background using a set of 30,000 randomers with similar GC distributions. Scanning and spot quantification of the arrays were performed using a Genepix 4000B scanner with GenePix 6.0 software. Each TFOE strain was analyzed with a minimum of three replicates. Mask alignment and robust multi-array average (RMA) normalization were performed after exporting data to NimbleScan software followed by subsequent statistical analysis and data visualization with Arraystar software. To compare against a baseline, median expression values were calculated for all genes across all 698 input microarrays. Altered gene expression was considered significant if it produced a moderated *t*-test *P*<0.01 after Benjamini-Hochberg multiple testing correction.

## Data Records

### Data record 1

All raw sequencing data for ChIP-seq experiments in BAM format are available at NCBI under BioProject number PRJNA255984 (Supplementary Table 1) (Data Citation 1). In addition, sorted and indexed BAM files are available at the MTB Network Portal (http://networks.systemsbiology.net/mtb/chipseq-gateway). The MTB Network Portal enables exploration of ChIP-seq data for each TF as UCSC Genome Browser Tracks and also provides download links for sorted BAM files. Binding events identified as described in Methods section are also presented along with associated transcriptional consequences.

### Data record 2

All transcription factor overexpression data from tiling microarray experiments are available at NCBI GEO database under the accession number GSE59086 (Supplementary Table 2) (Data Citation 2). Moreover, sample information for each experiment is available at MTB Network Portal (http://networks.systemsbiology.net/mtb/content/TFOE-Searchable-Data-File). A searchable Excel file enabling easy query of this large dataset can also be downloaded from the portal.

### Data record 3

Table summarizing TF binding locations for target genes from ChIP-seq experiments, expression levels of these genes in the corresponding TF overexpression tiling array experiments and overlap with regulatory network model is deposited into Figshare data repository as excel worksheet file (Supplementary Table 3) (Data Citation 3). This table is also available at MTB Network Portal (http://networks.systemsbiology.net/mtb/chipseq-gateway). Please note that this table is expanded version of Supplementary Table S3 in Minch *et al.* ^14^ study that includes overlap with regulatory network model from Peterson *et al.* ^16^ Description of the table columns is as follows; *Target Gene*: Gene with closest start to peak center, *Transcription Factor*: Transcription factor used in ChIP-seq and tiling array experiments, *ChIP Center*: the peak’s central location (unit=nucleotide position), *Distance*: Using ‘peak center,’ the nucleotide distance from the peak to the nearest start site, *Strand*: gene orientation of target on chromosome, *Genomic Position*: nucleotide coordinate of nearest start, Type: string description for category of proximal start site, *Expression*: log2 value describing differential expression of ‘gene’ after induction of the corresponding ‘regulator’ (See Rustad *et al.* ^15^), *Expression P-value*: *P*-value of this level of enrichment calculated by empirical Bayes (See Rustad *et al.* ^15^), *Differential Expression*: >1.5-fold change up (‘IND’) or down (‘REP’) of target gene upon induction of the corresponding regulator (See Rustad *et al.* ^15^), Operon: ID number for the operon target gene belong to, *Bicluster*: Bicluster ID from network model, *q.Bicluster*: number of genes in bicluster, *g.ChIPSeq*: number of genes that overlap the bicluster and ChIP-Seq target genes (from TF overexpression), *q.ChIPSeq.genes*: the gene IDs of q.ChIPSeq column, *q.DE*: number of genes that overlap the bicluster and differently expressed genes (from TF overexpression), *q.DE.genes*: the gene IDs of q.DE column.

## Technical Validation

### ChIP-seq control dataset

To determine significance thresholds for peak inclusion in our data set, we generated a ChIP-seq control compendium consisting of 10 different sequencing data sets as explained in Minch *et al.* ^14^ Because no single control type captures all known or potential ChIP artifacts or biases, we included an array of control types, including: wildtype H37Rv chromatin immunoprecipitated with and without anti-FLAG antibody, chromatin samples from uninduced expression vector-bearing cells immunopreciptated with and without anti-FLAG antibody, as well as chromatin samples from induced non-TF genes immunoprecipitated with anti-FLAG antibody. This control dataset was used to assign significance scores to called peaks. Peaks for all 10 control datasets along with associated scores were collapsed into a single data file to use as a negative control. Probabilities of having a similarly scored peak in the control dataset were calculated for each experimental peak and summarized in Supplementary Table 1 of Minch *et al.* ^14^

### ChIP-seq quality filters

We profiled the binding distribution for all TFs at different *P*-value cutoffs in order to determine high-confidence binding events. We found that, in general, p-values less than 0.05 showed stronger binding scores relative to negative control set and had lower signal to noise ratios. ‘Signal to noise ratio’ refers to relative enrichment of peaks for each experiment (Signal) over background peaks identified in the control datasets (Noise) considering background read-depth, distribution of forward and reverse strand reads, distribution of reads, and height-width ratios on each strand. Since binding events at *P*-values<0.05 were not consistently validated in our test, we only included peaks with *P*-values<0.01 for subsequent analysis (See Fig. 4 in Minch *et al.* ^14^). We further compared high-occupancy sites for any systematic biases for ChIP enrichment by comparing them to absolute log2 expression ratios from more than 700 TFOE experiments. This investigation did not find any correlation with ChIP enrichment and transcript abundance. As a conservative filter, we removed any region that included binding by more than 50 different TFs.

### Assessment of DNA-binding events with transcriptional regulation

**Figure 2:**
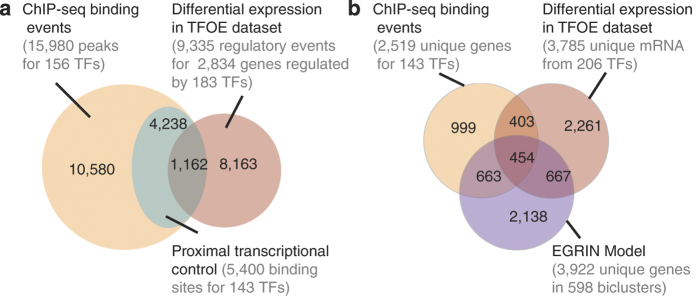
Comparison of ChIP-Seq, TFOE data and EGRIN model. (**a**) We investigated the overlap between ChIP-seq binding events^14^, differential expression in the TFOE dataset^15^ and proximal binding in promoter window analysis^14^ in order to assess transcriptional consequences of DNA-binding events. (**b**) ChIP-seq and TFOE datasets were further compared to regulatory influences identified in EGRIN model^16^ to validate data-driven model predictions with experimentally identified influences.

To determine the extent of TF-binding locations associated with direct regulation, we analyzed instances where TF overexpression resulted in significantly altered expression of genes proximal to TF binding sites. We first explored ChIP-seq binding locations relative to transcription start sites (TSSs) of annotated genes (Collected from Cortes *et al.* ^20^) and then determined that a consensus promoter region spanning 150 bp upstream to 70 bp downstream of TSSs is most enriched in binding events that are considered functional^14^. Thus, 5,400 binding sites for 143 TFs were located within the −150 to 70 bp promoter window of 7,248 genes and were considered to be capable of directly regulating downstream gene expression in the right environmental context. Expression analysis of TF overexpression in reference laboratory growth conditions validated 1,162 TF-DNA interactions that directly regulate proximal genes (Data Citation 3 and Fig. 2a). We were able to validate over 20% of all promoter-proximal binding events using only one growth condition, and considering the known conditional nature of gene regulation, an appropriate environmental context would likely validate regulation of a majority of the 7,248 DNA-binding locations in the presumed promoters of 2,520 unique genes.

### Comparison of TFOE results with existing datasets

We compared the overlap between 12 previously defined MTB regulons and TFOE-derived regulatory influences in order to evaluate our results. For each comparison, a hypergeometric *P*-value was calculated to identify statistically significant overlap between each set. The majority of TFOE-defined regulons overlapped significantly with those previously identified (Supplementary Table 1, and Table 1 in Rustad *et al.* ^15^). On average, the genes triggered by TF overexpression included 70% of genes in previously characterized regulons (*P*-value on average less than 0.001). In two-thirds of cases, the number of genes regulated by TFOE was substantially larger than the corresponding regulons described in the literature. Only two previously reported regulons, from Rv0195 and Rv2034, showed poor overlap with the TFOE dataset. Considering both are associated with the MTB Enduring Hypoxic Response, TFOE experiments performed under standard conditions may not trigger their activity.

## Usage Notes

The systems-scale studies described herein were designed to map the networks of gene regulation that underlie the adaptability of MTB. High-throughput experiments were performed to determine genome-wide TF binding and gene expression following induction of each TF. These datasets are essential to TB researchers addressing questions concerning the regulation of processes driving MTB pathogenicity. Here, we present the integration of these datasets with an independent gene regulatory network model of MTB, exploration using the MTB Network Portal (http://networks.systemsbiology.net/mtb/), and options for alternative data analyses.

### Integration with MTB gene regulatory network model

We used the TF overexpression datasets to assess the accuracy of an MTB environmental and gene regulatory inference network (EGRIN) model, published in Peterson *et al.* ^16^ The EGRIN model was trained on a compendium of 2,325 publically available microarray experiments to decipher a predictive transcriptional regulatory network model of MTB. The model identified sets of genes (modules) that are co-regulated under a subset of experimental conditions, have a common motif in their promoters and are enriched in protein–protein interactions. The resulting modules were organized into a network model of gene regulation. We tested whether the grouping of genes within modules of the model agreed with the distribution of TF-binding locations and gene expression changes from the TF overexpressing datasets. To integrate these data, we investigated how often two or more genes were simultaneously found within a module, bound by the same TF and differentially regulated upon over-expression of that TF. This analysis identified a set of 454 unique genes that were co-regulated in varying combinations across 240 modules by 57 TFs (Benjamini–Hochberg, BH, corrected permuted *P*-value<0.01, Fig. 2b, Data Citation 3). We also compared the genes in biclusters discovered by EGRIN to the experimentally characterized targets of every overexpressed TF. This comparison showed that the network model accurately recalled co-regulated genes for 41% of the overexpressed TFs (57 out of 140 at *P*-value≤0.05 for all TFs with ≥2 unique genes) and recovered 49% of the TF–gene interactions from the TF overexpression set (793 out of 1,635 genes that were both ChIP-Seq targets and differentially expressed upon over-expression of a TF). The 49% recovery rate was greater than validated interactions from other transcriptional regulation modeling algorithms using expression data^21,22^. Thus, we rigorously validated the co-regulation of genes across 240 modules in the MTB EGRIN model and substantiated the use of the TF overexpression datasets for mapping networks of gene regulation in MTB.

### Access and exploration of MTB gene regulation using the network portal

**Figure 3:**
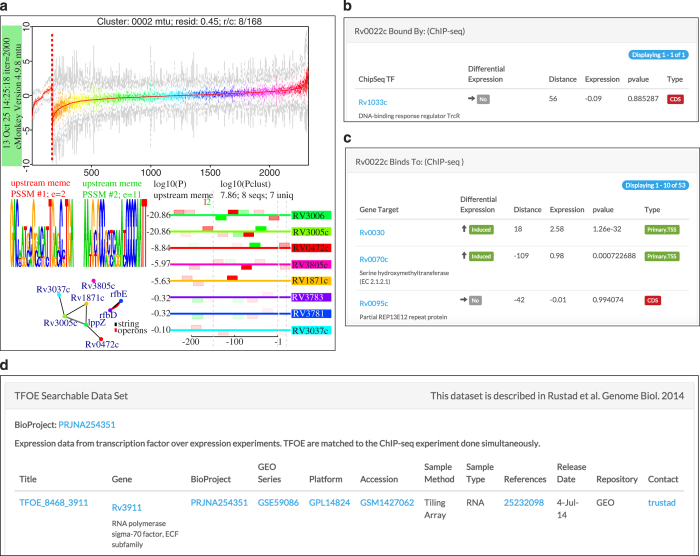
Screenshot of MTB Network Portal highlighting modules, binding events and dataset tables. MTB Network Portal provides gene- and regulatory module-centric visualizations and integrate with other TB resources such as Tuberculist and PATRIC. Only few example features are highlighted in here. (**a**) Expression profile of all the genes in the module together with *de-novo* identified motifs and motif locations are displayed for regulatory modules. (**b**) Table of TFs that bind with close proximity of a given gene from ChIP-seq experiments is listed on the gene page. (**c**) A table of ChIP-seq binding events with details for targets of a given TF is displayed together with expression consequences. (**d**) Detailed information for each dataset (ChIP-seq or TF overexpression) is given together with links to corresponding repositories and portal resources.

**Figure 4:**
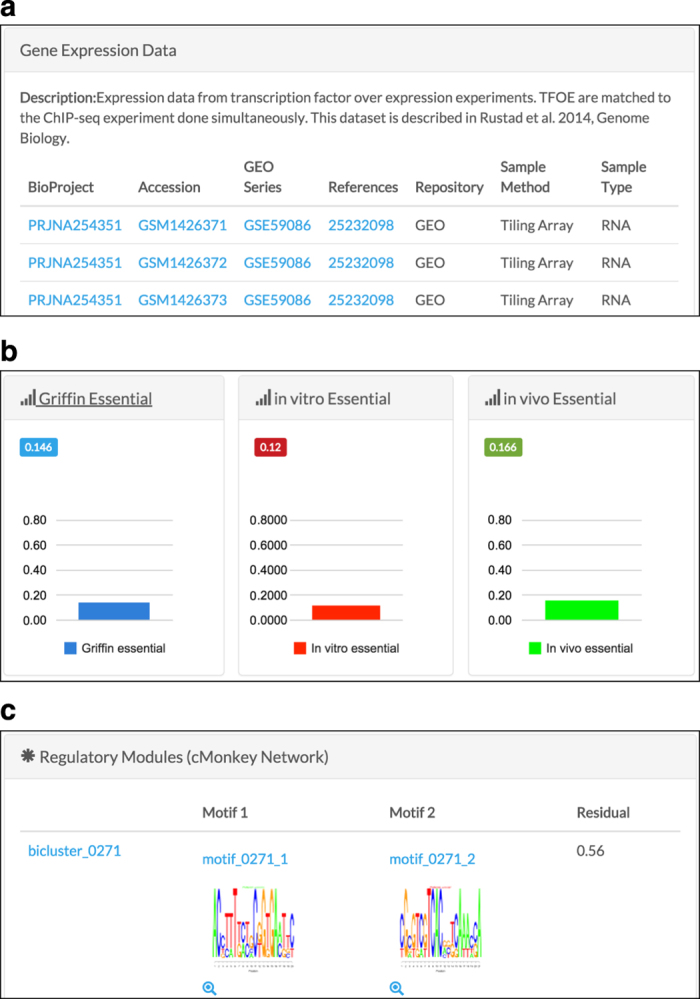
Screenshot of MTB Network Portal highlighting expression data, essentiality and regulatory modules. Gene detail pages include information for available experiments associated with given a gene, essentiality graphs and link to regulatory modules that contain this gene. (**a**) Available gene expression datasets from TF overexpression data are listed on the gene page for each TF. (**b**) *In vivo* and *in vitro* essentiality data is also shown on the gene page. (**c**) Regulatory modules that include the gene are displayed with residual, motif logos, and motif e-values.

To enable exploration of the TF overexpression datasets and EGRIN model, we have made all data accessible through a user-friendly web-portal (http://networks.systemsbiology.net/mtb/). The network portal serves as a modular database for the storage, visualization and analysis of the gene regulatory network data^23^. Importantly, the MTB network portal improves accessibility to the regulatory data by providing basic and advanced search interfaces, easy-to-use filters, and integration with established web-based resources (Figs 3 and 4).

Specifically, the network portal contains the ChIP-seq and expression data from TF overexpression experiments along with all ~600 EGRIN modules (Fig. 3). These data are linked at the gene level (that is, ‘name’, ‘locus tag’, or ‘function’) and are also connected to online resources, including NCBI GenBank^24^, UniProt^25^, Tuberculist^17^, PATRIC^26^ and TBDB^27^. Moreover, the gene regulatory network data are integrated with protein-protein interactions from EMBL STRING^28^, and functional enrichment from the Gene Ontology^29^, KEGG^30^, and BioCyc^31^. All this information can be retrieved by executing searches based on unique genomic, functional and network parameters as well as ranges or combinations of these values.

A search for any of the ~4,000 genes of MTB presents a gene-landing page with genomic, functional and regulatory information for individual genes. The gene page provides an overview of EGRIN module membership, motifs associated with these modules, functional ontology assignments, and links to the aforementioned online resources. If the gene is a transcription factor, TF binding sites identified by ChIP-seq are visualized with Circos plots^32^ that display connections between the TF and its target genes from ChIP-seq DNA-binding events. The gene page also lists ChIP-seq binding targets and includes proximal regulation, if any, as determined by microarray expression profiling. Links to the complete expression profiling data, available through GEO^33^, are also provided (Fig. 4). The organization and comprehensiveness of the MTB network portal makes it straightforward for the TB research community to access, explore, and analyze gene regulation of MTB.

### Alternatives for data analyses

For each step in the ChIP-seq analysis workflow, there are many software packages available. For example, peak callers employed in ChIP-seq analysis include: CSAR^34^, PeakRanger^35^, and SPP^36^. Similarly, there are many methods for example, ChIPDiff^37^, Comparitive ChIP-seq^38^, and POLYPHEMUS^39^, for analysis of differential binding in ChIP-seq. In addition, many publically available software packages, for example, limma^40^, RankProd^41^, and TIGR MultiExperiment Viewer (MeV) [http://www.tm4.org/mev.html], could be used for analysis of microarray expression data. While there are many alternatives for mapping, normalizing and quantifying the TF overexpression datasets, the processed data provided here has been peer-reviewed and successfully used to reveal biological insights into the gene regulation of MTB.

## Additional information

**How to cite this article:** Turkarslan, S. *et al.* A comprehensive map of genome-wide gene regulation in *Mycobacterium tuberculosis*. *Sci. Data* 2:150010 doi: 10.1038/sdata.2015.10 (2015).

## Supplementary Material



Supplementary Table 1

Supplementary Table 2

Supplementary Table 3
